# Human amniotic epithelial cells exert anti-cancer effects through secretion of immunomodulatory small extracellular vesicles (sEV)

**DOI:** 10.1186/s12935-022-02755-z

**Published:** 2022-10-29

**Authors:** Mohammad-Reza Bolouri, Roya Ghods, Kayhan Zarnani, Sedigheh Vafaei, Reza Falak, Amir-Hassan Zarnani

**Affiliations:** 1 Department of Immunology, Iran University of Medical Sciences, Tehran, Iran; 2 Oncopathology Research Center, Iran University of Medical Sciences, Tehran, Iran; 3 Department of Molecular Medicine, Faculty of Advanced Technologies in Medicine, Iran University of Medical Sciences, Tehran, Iran; 4 Department of Immunology, School of Public Health, Tehran University of Medical Sciences, Tehran, 1417613151 Iran; 5 Biotechnology Research Center, Avicenna Research Institute, ACECR, Tehran, Iran; 6 Immunology Research Center, Iran University of Medical Sciences, Tehran, 1449614535 Iran

**Keywords:** Stem cells, Human amniotic epithelial cell, Cancer, Small extracellular vesicles (sEV), Cytotoxic T lymphocyte, Warburg’s effect, Metabolomics

## Abstract

We identified here mechanism by which hAECs exert their anti-cancer effects. We showed that vaccination with live hAEC conferred effective protection against murine colon cancer and melanoma but not against breast cancer in an orthotopic cancer cell inoculation model. hAEC induced strong cross-reactive antibody response to CT26 cells, but not against B16F10 and 4T1 cells. Neither heterotopic injection of tumor cells in AEC-vaccinated mice nor vaccination with hAEC lysate conferred protection against melanoma or colon cancer. Nano-sized AEC-derived small-extracellular vesicles (sEV) (AD-sEV) induced apoptosis in CT26 cells and inhibited their proliferation. Co-administration of AD-sEV with tumor cells substantially inhibited tumor development and increased CTL responses in vaccinated mice. AD-sEV triggered the Warburg’s effect leading to Arginine consumption and cancer cell apoptosis. Our results clearly showed that it is AD-sEV but not the cross-reactive immune responses against tumor cells that mediate inhibitory effects of hAEC on cancer development. Our results highlight the potential anti-cancer effects of extracellular vesicles derived from hAEC.

## Highlights

- Anti-cancer effects of hAEC depend on cancer type.
- Cross-reactive humoral responses do not mediate anti-cancer effects of hAEC.
- Anti-cancer effects of hAECs are mainly mediated by small-extracellular vesicles (sEV).
- hAEC-derived sEV (AD-sEV) trigger the Warburg’s effect leading to Arginine consumption and cancer cell apoptosis.
- AD-sEV substantially inhibits tumor development and increases survival and CTL responses.

## Background

Cancer is a leading cause of death and a major public health problem worldwide [1] pointing to the priority of studies in this field. Efforts for the development of an effective treatment have led to several cancer therapy approaches. However, in most cases, the effectiveness of therapeutic modalities is far from our expectation, which mainly stems from the diagnosis of the tumor at late stages. In this regard, finding robust cancer preventive and therapeutic approaches has been the focus of many researchers.

The immunologic similarity between cancer and pregnancy was proposed as early as 1884 [2] and motivated many researches in the field. Immunization of animals with embryonic materials induces strong cellular and humoral immune responses against tumors [3] supporting the concept of usefulness of targeting embryonic cross-reactive antigens to stimulate anti-cancer immune responses. After decades of scientific gap, this concept re-emerged in a more fascinating form, the cancer stem cell theory [4]. Recent studies have shown ectopic expression of such embryonic antigens as SSEA-3, SSEA-4, Oct-4, and Nanog, in cancer cells [5–7] highlighting the potential of embryonic-derived antigens to be employed in cancer vaccines [8, 9]. There are several reports on the potential efficacy of embryonic stem cells (ESCs) on the induction of cross-protective immune responses against murine colon carcinoma [10] and lung cancer [11, 12]. However, ethical concerns have also been a major obstacle to using fetal materials for tumor immunity. This limitation led investigations to a more ethically acceptable source of embryonic origin. The Placenta is a unique organ existing for a short period in the body during gestation and is fundamental for appropriate fetal development. It is a site for the expression of many antigens and molecular markers shared by many cancer cell types [13–16] and hosts a collection of cells with stem cell properties [17]. Human amniotic epithelial cells (hAEC) are among placenta-derived cells with known stem cell-like and immunomodulatory properties [18–20]. In this regard, immunosuppressive and anti-inflammatory properties of hAEC have been the main objective of several studies [21–25]. Nonetheless, anti-cancer effects of hAEC have also been the focus of recent investigations. In a therapeutic perspective, hAEC showed remarkable anti-tumor effects in breast cancer-bearing nude mice [26]. Vaccination of mice with hAEC inhibited the formation of colon cancer in a mouse model of CT26 colon cancer [10]. The authors suggested that the cross-reactive humoral and cellular immune responses could confer protective immunity against colon cancer in mice vaccinated with human hAEC [10]. In a nude mouse model of ovarian cancer, hAECs significantly decreased the average volume and weight of xenografted tumors. GFP-labeled hAEC was found in the stromal area of xenografted tumor tissues 28 days post injection. The authors also reported higher expression of three negative regulators of cell cycle progression, p16^INK4A^ and p21, and phospho-JNK in the tumor tissues of hAEC vaccinated mice [27]**.** Besides a direct negative impact on tumor cells growth, hAECs also exert substantial anti-tumor effects through their secretome. Conditioned media (CM) of hAEC induced apoptosis of breast cancer cells and showed anti-angiogenic effects [28–30]. hAEC CM also induces G0/G1 cell cycle arrest and inhibits the division of ovarian cancer cells. hAEC secretome contained several anti-cancer-related cytokines,including TGF-β1 with capacity to negatively regulate cell cycle progression [27]. In another study, CM of rat derived-AEC (rAEC) dose-dependently inhibited the proliferation of B16F10 and HepG2 tumor cells [31]. Tumor size in mice that received B16F10 cells treated with CM of rAEC was restricted in a dose-dependent manner and even no tumor development was observed in mice treated with 100% CM of rAEC [31]. These results suggest that hAEC is endowed with a secretory component that is capable to inhibit the functionality and proliferation of cancer cells.

Recently, the potential of extracellular vesicles in modulating cancer cell behavior has been highlighted. A growing body of evidence suggests that extracellular vesicles and their cargos serve as a therapeutic modality, cancer prognostic marker, or even as anti-cancer drug‐carrier [32]. Accordingly, some recent studies have demonstrated the role of extracellular vesicles in hAEC function, triggering the idea that some of the mentioned anti-cancer effects of hAECs could be due to extracellular vesicles components [33]. Extracellular vesicles are extracellular nanovesicles, which are produced and secreted by most of eukaryotic cells to communicate with their environments. These vesicles range from 30 to120 nm in size and contain several cellular components including DNA, RNA, proteins, lipids, and other substances which could be absorbed into the target cell and lead to some functional alterations [33–38]. Interestingly, the functions reported for hAEC derived-small extracellular vesicles (AD-sEV) and hAEC cells are highly overlapping. For example, the protective effect of hAEC on the development of lung fibrosis through immune suppression mechanisms in a mouse model of bleomycin-induced fibrosis [34] is similar to the effects of (AD-sEV) [39]. Additionally, several other studies on AD-sEV confirmed their immunomodulatory effects, comparable to the previously obtained results from intact hAEC [34, 37, 40–42].

Antigen similarity and induction of cross-protective immune responses have been proposed as one potential mechanism of anti-cancer action of hAEC. Here, we tested using different immunization systems to explore to which extent this mechanism is responsible for anti-tumor activity of hAECs and explored for the first time the potential anti-cancer mechanism vaccine effect of hAECs in mouse models of colon, breast and melanoma cancers.

## Materials and methods

### Animals, cell lines and tissues

Female 6–8-week BALB/c and C57BL/6 mice and all cell lines used in this study were purchased from the Pasture Institute of Iran. CT26 and 4T1 cells were used for the induction of colon and breast cancers, respectively, in BALB/c mice, while B16F10 was used to induce melanoma in C57BL/6 mice. 3T3 normal mouse fibroblast and MCF7 human breast cancer cells were used as control cells in some settings. Cells were cultured in RPMI-1640 supplemented with 10% fetal bovine serum (FBS) and antibiotics and incubated in a CO2 incubator at 37 °C. Animals were kept in standard condition with a 12 h light–dark cycle and fed ad libitum. Amniotic membranes were obtained from healthy pregnant women delivered by elective cesarean. All women signed an informed consent form before participation in this study. All procedures conducted in this study, including animal experiments were approved by the ethics committee of the Iran University of Medical Sciences, IUMS (IR.IUMS.REC.1395.28042).

### Reagents and antibodies

Most of the reagents used in cell culture were obtained from Gibco (UK) unless specifically indicated. The primary and secondary antibodies were as follows: Rat anti-SSEA-3 (Invitrogen, USA), phycoerythrin (PE)-SSEA-4 (eBioscience, USA), mouse anti-TRA1-60 (Millipore, USA), PE-OCT-4 (BD Pharmingen, USA), rabbit anti-Nanog (Abcam, USA), fluorescein isothiocyanate (FITC)-goat anti-rabbit Ig (Abcam,), FITC-goat anti-mouse (Biorad, USA), FITC-conjugated sheep anti-mouse Ig (Sina biotech, Iran), and HRP-conjugated sheep anti-mouse Ig (Sina biotech). Extracellular vesicles-specific antibodies were from SBI system biosciences, CA, USA. Extracellular vesicle-depleted fetal bovine serum was purchased from Gibco. PKH-26 dye labeling kit was from Sigma (USA). Annexin V/PI apoptosis kit, ECL, BCA protein assay kit and calcein**-**acetoxymethyl (cAM) were purchased from ebioscience (USA), GE healthcare (USA) and BD Pharmingen, respectively.

### Isolation and characterization of human amniotic epithelial cells

Human amniotic epithelial cells (hAEC) were isolated from amniotic membranes obtained from term pregnancies delivered by elective cesarean section from healthy women aged 20 to 40 years. In brief, amniotic membranes were mechanically peeled away from the underlying chorion, washed several times with cold phosphate buffered saline (PBS) and digested in 0.05% trypsin–EDTA buffer for three steps. Single cell suspensions obtained from the second or third steps were pooled and analyzed by flow cytometry for surface antigens, SSEA-3, SSEA-4, Nanog, TRA-1–60, and OCT-4. For immunofluorescent staining, isolated cells were fixed in ice-cold acetone and stained with FITC-conjugated antihuman cytokeratin (BD, USA). Vimentin staining of fixed hAECs was performed with the addition of 5 µg/mL mouse anti-human vimentin antibody (Santa Cruz, USA) followed by FITC-conjugated sheep anti-mouse immunoglobulin (Sina Biotech). DAPI (Sigma) with a final concentration of 2 µg/mL was used for nuclear staining.

### Culture of human amniotic epithelial cells

Isolated hAECs were cultured in complete media, high glucose Dulbecco’s modified Eagle’s medium (DMEM) supplemented with 10% FBS, antibiotics and 10 ng/mL epidermal growth factor (EGF, Royan Institute, Tehran, Iran) and incubated in a humidified CO2 incubator at 37 °C. After 24 h, non-adherent cells were removed and fresh medium was added to adherent cells and incubation was continued for 48 h. Then, cells were dissociated with 0.25% trypsin–EDTA solution and washed with PBS and used for immunization.

### Extracellular vesicles isolation and characterization

Isolation of hAEC-derived extracellular vesicles (AD-sEV) was performed as described previously [35]. Briefly, hAECs were cultured in complete media containing 10% exosome-free FBS and 10 ng/mL EGF until they reached approximately 90% confluency. The conditioned medium was collected and centrifuged at 1000 × *g* (20 min, 4 °C) and then filtered through 0.22 μm filter to remove cell debris. Then, the prepared CM was ultracentrifuged for 70 min at 100,000 × *g*, 4 ºC and the supernatant (extracellular vesicle-free condition medium) was removed and the pellet was re-suspended in PBS and centrifuged again under the same conditions. Finally, the pellet was re-suspended in an appropriate volume of PBS and stored at – 80 ºC as AD-sEV.

To determine extracellular vesicle size, freshly-isolated extracellular vesicles were diluted in PBS and their size was determined by Zetasizer (Nano ZS, Malvern Instruments, UK) at ambient temperature of 23–28 °C. The measurement was performed at least thrice in three independent experiments. The Protein concentration of isolated extracellular vesicles was determined by BCA assay according to the manufacture’s recommendation. Western blotting was performed for CD81 and CD63 expression in isolated extracellular vesicles. The western blot was performed as described in the SBI Systems Bioscineces exosome antibodies kit. Briefly, 50 µg protein per lane was added to the sample buffer (containing 5% 2-mercapto ethanol), boiled (5 min), and subjected to SDS-PAGE (5% stacking and 12.5% resolving) electrophoresis at 100 V for 70 min. The proteins were transferred onto polyvinylidene fluoride (PVDF) membrane (Roche, USA). The membrane was blocked for 1 h with 5% skim milk in Tris-buffered saline (TBS, pH = 7.5). After washing with TBS containing 0.05%Tween-20 (TBST), membrane was incubated overnight at 4 °C with extracellular vesicles primary antibody diluted 1:1000 in 5% skim milk in TBST. After washing three times in TBST, the membrane was incubated with secondary antibody (goat anti-rabbit-HRP) 1:20,000 for 1 h at room temperature. After washing, signals were developed with ECL (GE,UK) and visualized on film (Kodak, Japan).

### Extracellular vesicle labeling

The purified AD-sEV was labeled using a PKH-26 red fluorescent cell linker kit (Sigma). Briefly, extracellular vesicles were re-suspended in 1 mL of diluent solution provided in the kit. Then, the labeling solution was prepared according to the kit manual, mixed with an equal volume of diluted extracellular vesicles, and incubated for 10 min with periodic mixing. The labeling was stopped by adding an equal volume (2 mL) of 1% BSA and waiting for 1 min to allow binding and neutralization of the excess dye to the added protein. Labeled extracellular vesicles were washed with PBS and collected using ultracentrifugation (100,000 × *g* for 90 min). Labeled AD-sEV were diluted in PBS based on their protein content obtained from the BCA assay.

### Extracellular vesicles uptake assay

CT26 cells were seeded in 24 well plate until they reached 60–70% confluency. Then, labeled AD-sEV were added to each well (10 µg/ml). After 2, 3, 4, and 24 h, the cells were assessed by an inverted fluorescence microscope (Olympus BX51, Japan) equipped with a DP71 CCD camera to determine extracellular vesicle uptake.

### Cytotoxicity assay

An MTT assay was used to evaluate the cytotoxic effects of AD-sEV on cancer cell lines. In brief, 2 × 10^3^ cancer cells were cultured in each well of 96-well culture plates in a volume of 100 µL and titrating concentrations of AD-sEV from 2.5 to 10 μg/mL were added to the wells. After 48 and 72 h incubation, 20 μl MTT solution (5 mg/mL) was added to each well and incubated for three h at 37 °C. Then, the supernatant was removed and the formazan crystals were dissolved by adding 100-μl dimethyl sulfoxide (DMSO) and the plate was incubated for 10 min on a shaker at 37 °C. Control wells received culture medium instead of AD-sEV. The absorption of the wells was measured at 570 nm using an ELISA microplate reader (Biohit, BP 800, Finland).

### Apoptosis assay

For evaluation of potential apoptotic effects of AD-sEV on cancer cell lines, cells were cultured in 24-well plates at a density of 7000/well in a volume of 300 µL and treated with AD-sEV as above. The extent of apoptosis was measured by flow cytometry using Annexin-V/PI apoptosis detection kit (Biolegend, San Diego, CA, USA) according to the manufacturer’s instruction.

### Measurement of cellular glucose consumption

3 × 10^4^ CT26 was cultured in each well of 24 well culture plates in a final volume of 350 µL RPMI containing 3% FCS. After overnight culture, culture media were removed and substituted with the same medium containing 5, 10, or 20 µg/mL AD-sEV. Untreated CT26 and culture media treated with the same concentrations of AD-sEV served as controls. All treatments were done in triplicate. Cell culture was continued for 72 h. After 48 h and 72 h, a sample of 60 µL was collected from each well, centrifuged and used for metabolomics study. The remaining cell culture supernatants in the wells were collected after 72 h for the measurement of glucose and lactate. The cell number in each well and the percentage of cell viability were measured by trypan blue exclusion. Glucose and lactate concentrations in the media were measured using the glucose and lactate colorimetric assay kits (GLUC3 and LAC2, both from Cobas) according to the manufacturer’s directions.

### Amino acid metabolomics analysis

Amino acid concentrations in cell culture supernatants were measured using a MS/MS system (Qsight 210 MD, Perkin Elmer, and USA). Neobase non-derivatized standards used in this study were from Perkin Elmer (Finland).

### Immunization and tumor challenge

Each group of immunization consisted of at least 6 mice. For the basic vaccination protocol, mice were subcutaneously immunized with 1 × 10^6^ live adherent hAEC and boosted twice every other week. In parallel, control groups received 100 µL PBS, subcutaneously. In some settings, hAEC lysate was used for mice vaccinations. Lysate was prepared using either RIPA buffer (Santa Cruz) or a physical procedures (freeze–thaw). The hAEC were trypsinized, washed twice with PBS and lysed by the addition of 1-mL of RIPA buffer to 2 × 10^7^ cells for 15 min on ice with intermittent vortexing. For physical lysis, 2 × 10^7^ cells were suspended in 1 mL PBS and lysed by successive freeze (− 80 °C) thaw (37 °C) cycles for five times. Lysates were then centrifuged at 12,000 *g* for 15 min and the supernatants were aliquoted and stored at − 80 °C. The protein concentration of the lysates was evaluated by the BCA method. Mice were immunized subcutaneously with hACE lysate prepared by either RIPA buffer or a mechanical procedure. Immunization was performed twice with 1 week interval. For each immunization, lysate from 10^6^ hAEC cells, CT26, or B16F10 was mixed with CpG 1826 (3 μg) and Poly I:C (25 μg) in 100 μL. PBS containing CpG and Poly I:C was used in the control group. One week after the last vaccination, mice were inoculated orthotopically (near the immunization region) or heterotopically (opposite site of the immunization region) with 5 × 10^5^ CT26, 10^5^ 4T1, or 10^5^ B16F10 live cells. Furthermore, to see the potential preventive effects of AD-sEV on tumor growth, tumor cells (5 × 10^5^ CT26, 10^5^ 4T1, or B16F10) were first pre-incubated with AD-sEV for 2 h at 37 °C, 5% CO_2_ incubator before injecting into the mice.

To test the potential therapeutic effects of AD-sEV, mice were challenged subcutaneously with the above-mentioned cell lines. AD-sEV (100 µL of 10 µg/mL) was injected into the tumor site after tumors being palpable for every 3 days for two weeks (totally six injections).

### Evaluation of tumor size

Tumor growth was monitored every three days using digital calipers to measure length (L) and width (W) of the tumors. The tumor area (L × W) was then calculated in mm^2^. Moreover, the mice were followed for their general health symptoms including behavior, feeding and body weight. The mice were euthanized when one dimension of a tumor reached 15 mm or the tumor area exceeded 225 mm^2^.

### Analysis of cross-reactive antibody responses by immunofluorescent staining

Cross-reactive antibody responses against CT26 and B16F10 cells in mice receiving hAEC were tested by immunofluorescent staining as described before [10]. Briefly, 1:200 dilution of mouse sera immunized with hAEC was incubated for 90 min with ice-cold acetone-fixed CT26, B16F10, or hAEC cells. In the negative control slides, primary antibody was substituted with the same dilution as non-immune mouse serum. After washing, slides were incubated cells with 1:50 dilution of FITC-conjugated sheep anti-mouse Ig (Sina Biotech, Iran) for 45 min. The nuclei were stained with DAPI. Signals were examined under a fluorescence microscope (Olympus BX51, Japan) equipped with a DP71 CCD camera. In some settings, the reactivity of hAEC-immunized mice was tested in MCF7 cells.

### Measurement of cytotoxic T lymphocytes response

To evaluate the effect of AD-sEV on cytotoxic T lymphocyte (CTL) response against tumor cells, quantitative cAM cytotoxicity assay was performed. Briefly, 2 weeks after the last injection of mice with CT26 (control group) or AD-sEV-treated CT26 cells (experimental group), spleens were removed and mononuclear cells were isolated by Ficoll density gradient. Splenocytes were then washed twice with PBS and their viability was assessed with trypan blue exclusion dye to ensure their viability was greater than 95%. Splenocytes, as effector cells, were added to the flat‐button 96‐well culture plates already seeded with 2 × 10^3^ CT26 cells/well at different effector-to-target cell ratios (50:1, 25:1, 12.5:1). After 72 h, the wells were washed twice with warm PBS and then 100 μL cAM (5 μM) was added to each well and the plates were incubated in a CO2 incubator for 30 min. Wells containing only CT26 or splenocytes served as positive and negative control wells, respectively. All experiments were performed in four replicas. The extent of fluorescence intensity was then measured by 1420 Multi-label fluorimeter counter (PerkinElmer, USA) with excitation and emission wavelengths of 485 and 535 nm, respectively. Percentage of cell cytotoxicity was calculated for each effector-target ratio using the following formula:

$$\% \,{\text{cytotoxicity}} = 1 - \left[ {\left( {{\text{Corrected}}\,{\text{mean}}\,{\text{fluorescent}}\,{\text{of}}\,{\text{test}}} \right)/\left( {{\text{Corrected}}\,{\text{mean}}\,{\text{fluorscent}}\,{\text{of}}\,{\text{control}}} \right) \times 100} \right],$$

where corrected mean fluorescent was calculated as the fluorescent readout of each well subtracted from average fluorescent readout of wells containing only splenocytes.

### Evaluation of T-cell frequency

Four groups were immunized with PBS, AD-sEV, CT26, or AD-sEV-treated CT26 as described above. Two weeks after immunization, spleens were removed and splenocytes were separated as described above. Finally, the percentage of CD4^+^ and CD8^+^ T cells was determined by flow cytometry (Partec, Germany).

### Statistical analysis

Statistical analysis was performed using GraphPad Prism 6.0 (GraphPad Software, Inc., La Jolla, CA, http://www.graphpad.com) software. The results were expressed as mean ± SEM. Comparisons between groups were done by Kruskal–Wallis and Mann–Whitney tests. P values less than 0.05 were considered as significant.

## Results

### Human amniotic epithelial cells express markers of embryonic origin

**Fig. 1:**
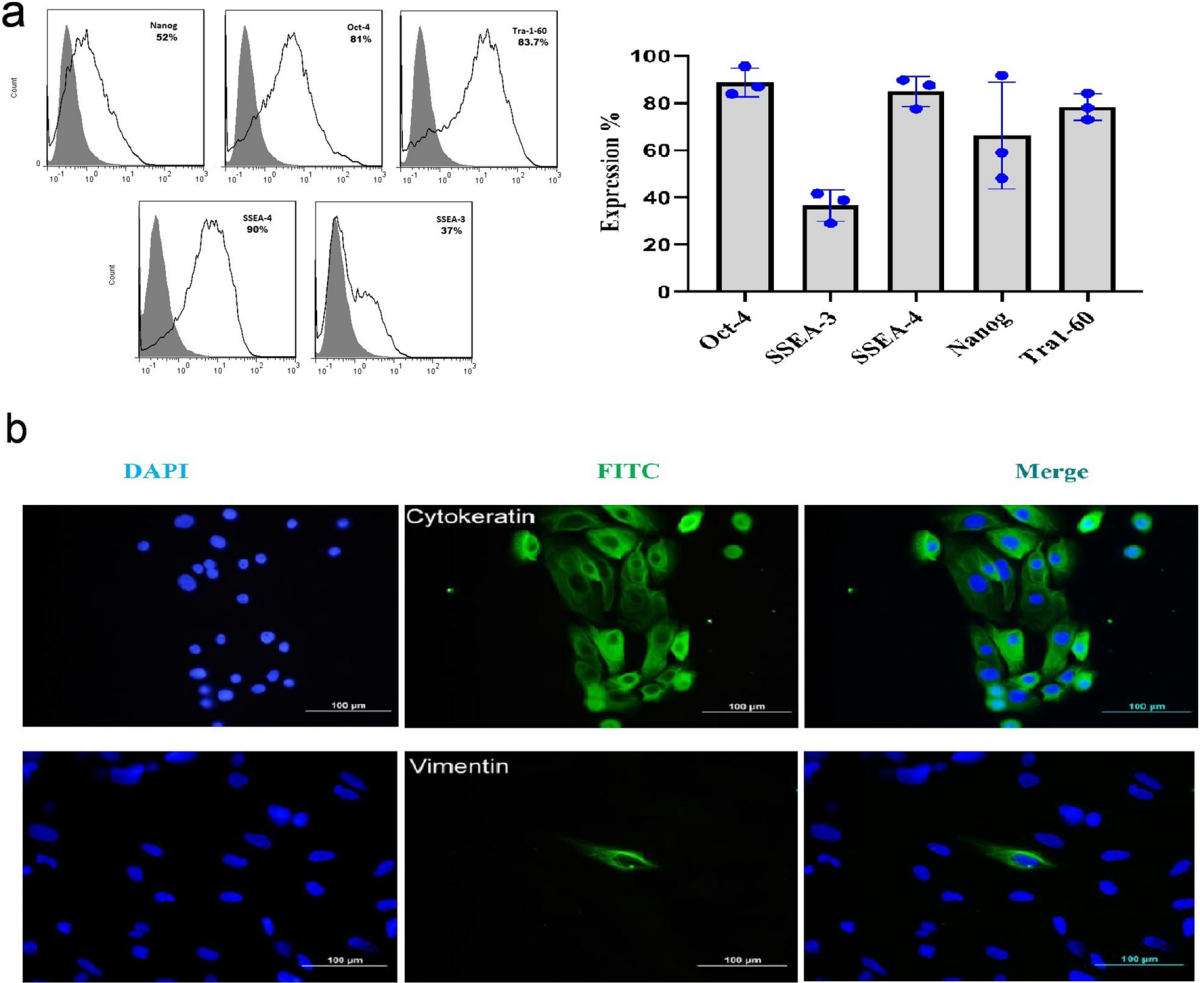
Human amniotic epithelial cells express embryonic related-markers. Expression of embryonic stem cell markers was assessed by flow cytometry (**a**) and quantified (**b**). The results are representative of three independent experiments. **c** Isolated human amniotic epithelial cells expressed cytokeratin, but failed to express vimentin

Amniotic epithelial cells were isolated from human amniotic membranes and characterized. About 100–250 × 10^6^ hAEC with viability of ≥ 90% were isolated from each membrane with high purity (≥ 90%) as judged by cytokeratin and vimentin staining. These cells appeared as flat, round cells with abundant cytoplasm and high cytoplasm to nucleus ratio. They expressed cytokeratin but failed to express vimentin (Fig. 1b). Immunophenotyping of hAECs was performed using flow cytometry. Accordingly, hAECs expressed embryonic stem cell markers, SSEA-3 (37% ± 3%), Nanog (52% ± 4%), TRA1-60 (83% ± 6%), SSEA-4 (90% ± 5%), and OCT-4 (81% ± 4%) (Fig. 1a).

### Vaccination with hAEC conferred protection and prolonged survival in murine models of colon cancer and melanoma but not in breast cancer

**Fig. 2:**
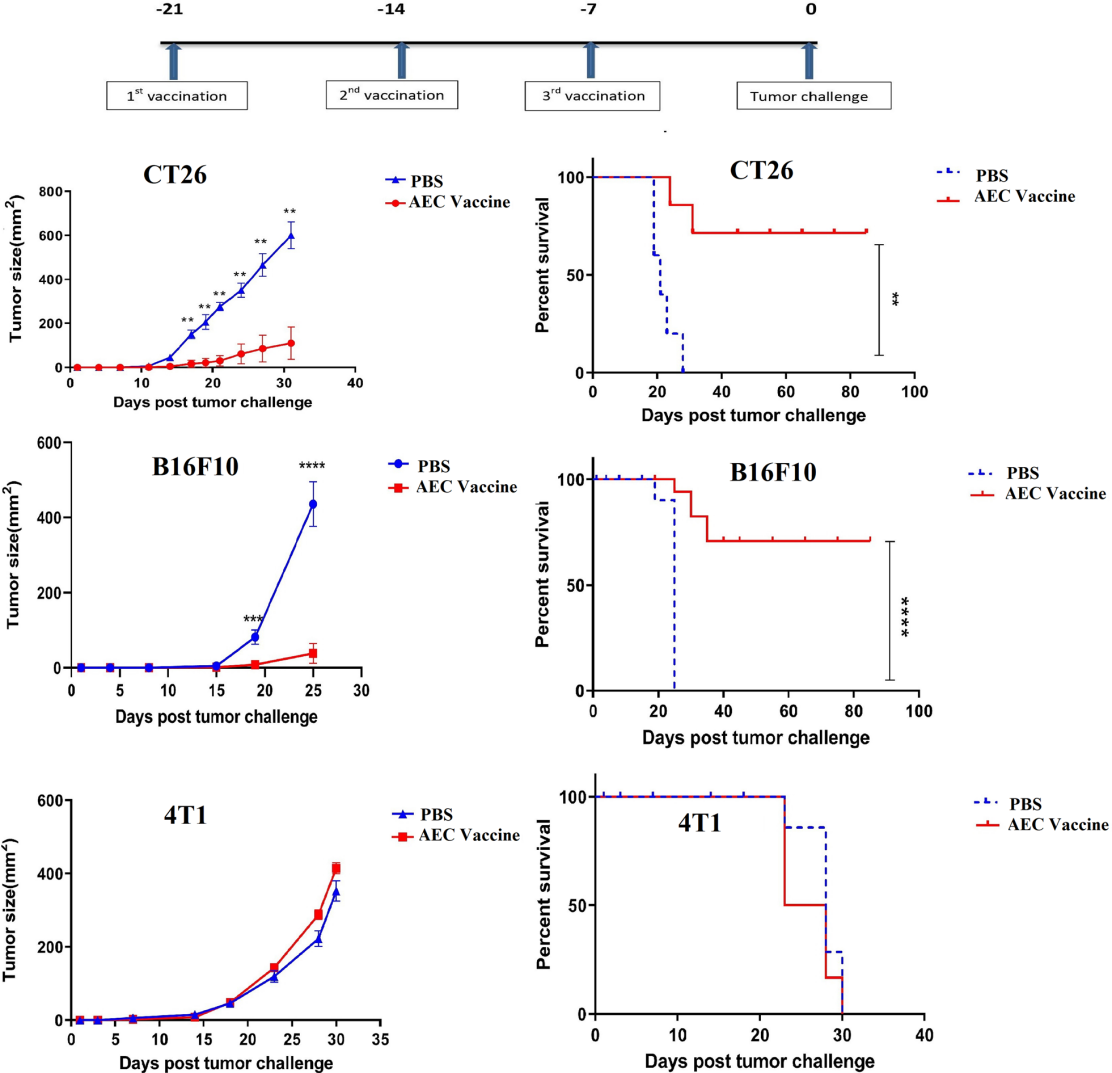
Vaccination with hAEC conferred protection and prolonged survival in murine models of colon cancer and melanoma but not in breast cancer. The top panel shows vaccination and tumor challenge scheme. 1 × 10^6^ hAEC were injected subcutaneously into BALB/c (n = 6) and C57BL/6 mice (n = 10) in phosphate-buffered saline three times at 1 week interval. The control mice received only PBS. Mice were challenged seven days after the last vaccination with 5 × 10^5^ CT26, 10^5^ 4T1 (BALB/c mice) or 10^5^ B16F10 cells (C57BL/6 mice) in the same side as the vaccination side. Tumor volumes were regularly monitored and calculated by measuring tumor dimensions (L × W) with digital calipers. In the case of survival rate, mice were considered dead when tumor surface area exceeded 225 mm^2^. Error bars denote mean ± SEM. Asterisks (*) indicate statistical significant differences (**p < 0.01, ****p < 0.0001). hAEC: human amniotic epithelial cells, PBS: Phosphate-buffered saline

To evaluate the effectiveness of hAEC vaccination in cancer protection, established models of colon, melanoma and breast cancers using CT26, B16F10, and 4T1 cell lines were employed. Mice were immunized with live hAEC or PBS (as control) every other week for three times and tumor induction was conducted 1 week after the last immunization (Fig. 2). After tumor induction, tumor size was followed up to 30 days. Remarkably, hAEC vaccination inhibited orthotopic development of colon and melanoma tumors in 83% (9 out 12) and 60% (6 out 10) of the vaccinated mice compared with the control group (Fig. 2). In those mice that developed tumor, the size of tumors was significantly less than that of the control mice. Vaccination with hAEC, however, exerted no beneficial effect on breast cancer development (Fig. 2). Vaccination also caused long-term survival (> 80 days) in vaccinated mice in colon cancer (p ˂ 0.01) and melanoma (p < 0.001) but not in breast cancer.

### Generation of cross reacting antibodies is dependent on cancer cell origin

**Fig. 3:**
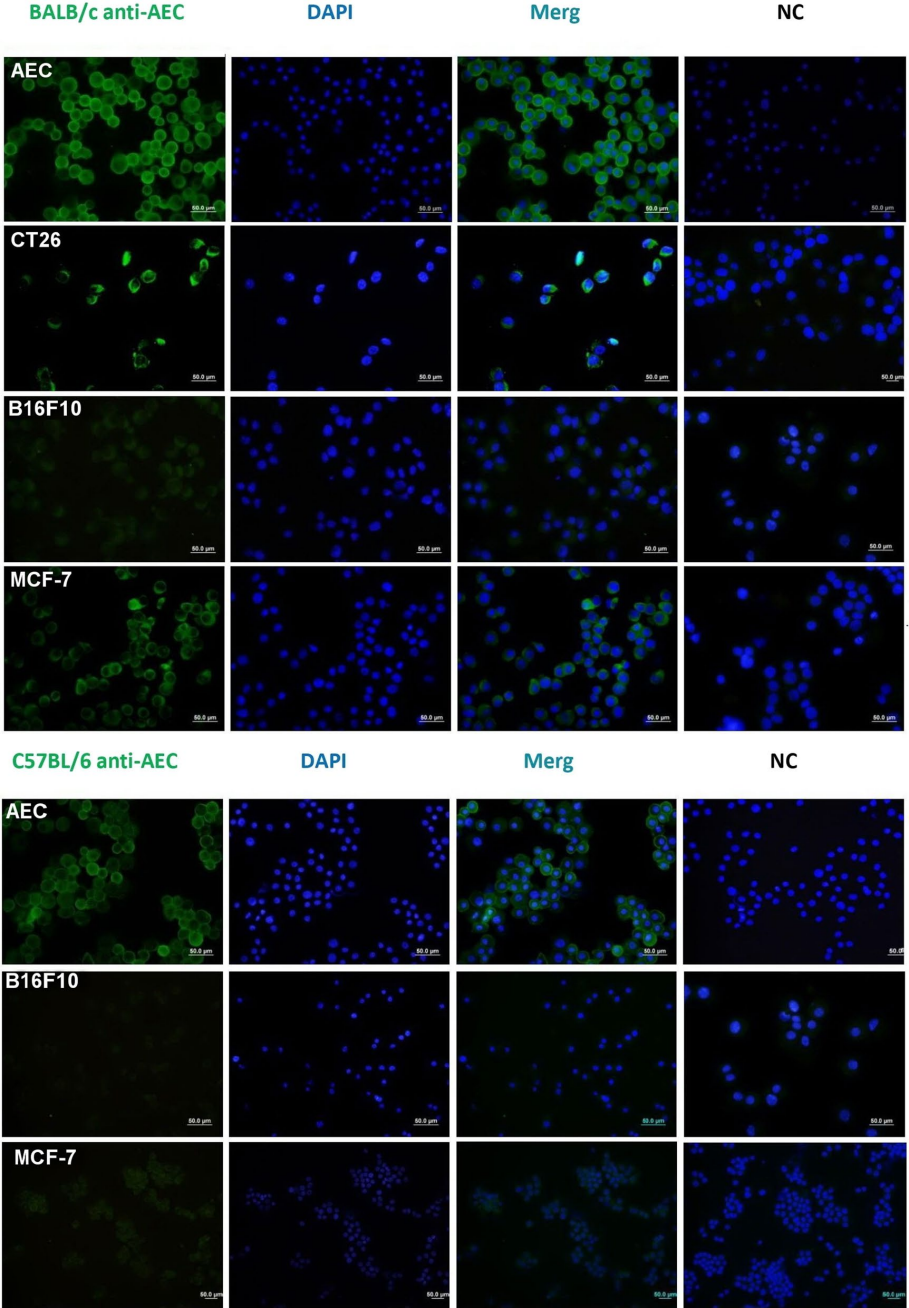
Immunization with hAEC generate cross-reactive antibodies against cancer cells. BALAB/c and C57BL/6 mice were immunized with hAEC thrice. Hyperimmune sera were collected and their reactivity was tested against immunizing hAEC and mouse cancer cells by immunofluorescent staining. Immunization of BALAB/c and C57BL/6 mice with hAEC generated cross-reactive antibody responses against cancer cells that depended on the cancer cell origin

Sera from Balb/C mice vaccinated with hAEC sharply reacted with hAECs and cross-reacted with CT26 and MCF7 cells with surface staining pattern but failed to react with B16F10 cells. The control mice sera reacted neither with hAEC, CT26 nor MCF7. Similarly, sera of hAEC-immunized C57BL6 mice reacted with hAEC but exhibited no cross-reactivity with B16F10 (Fig. 3).

### Vaccination with hAEC followed by heterotopic tumor induction neither conferred protection nor prolonged survival rate in a murine model of colon cancer

**Fig. 4:**
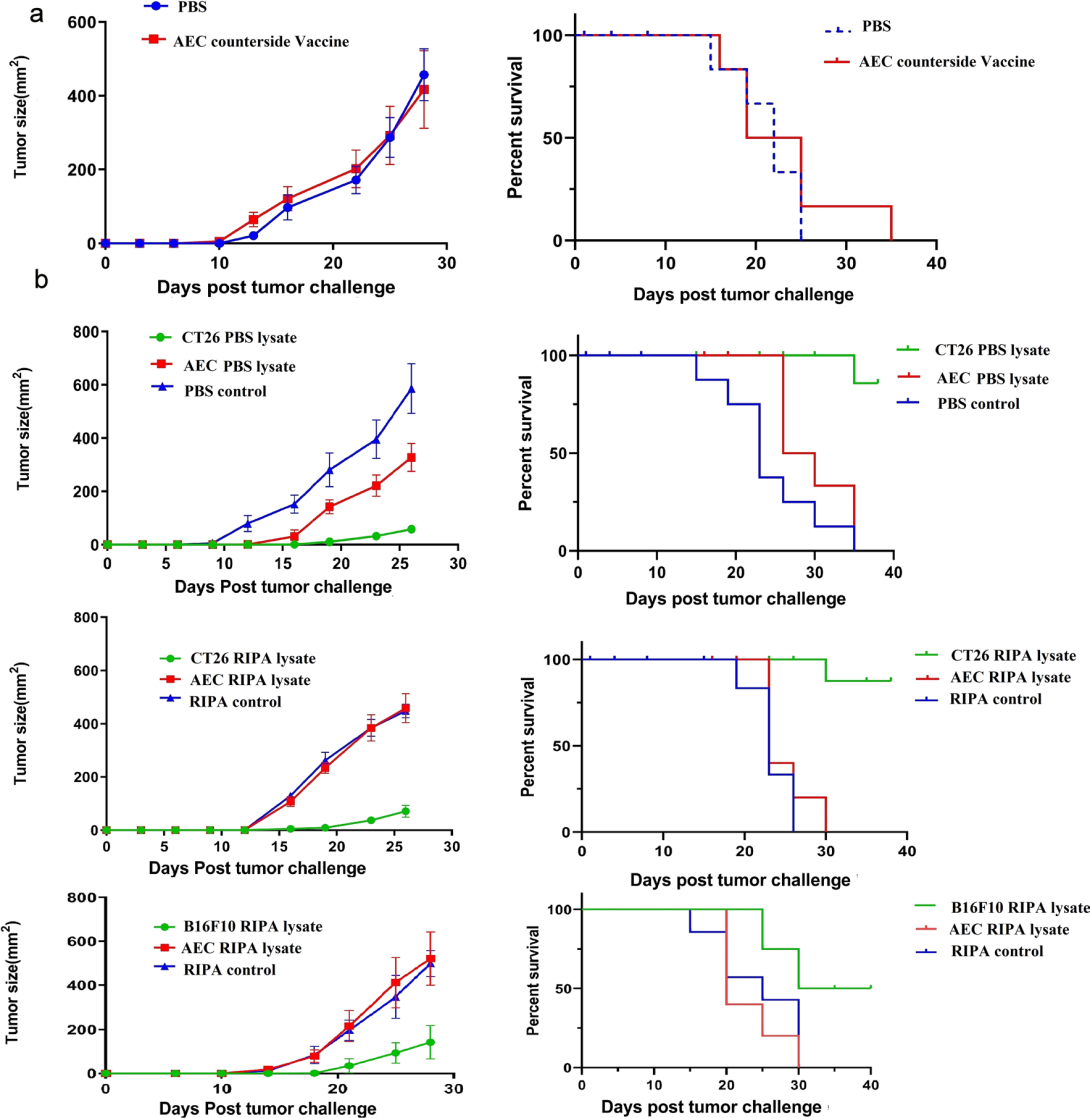
Immunization with hAEC lysate or heterotopic live hAEC vaccination neither conferred protection nor prolonged survival rate in a murine model of colon cancer and melanoma. BALAB/c mice were vaccinated in the right dorsal flank with 1 × 10^6^ hAEC for three times. Seven days after the last vaccination, mice were challenged with 5 × 10^5^ CT26 injected subcutaneously at the counter side of the vaccinations (left dorsal flank) (**a**). In other settings, BALAB/c mice were immunized twice with either hAEC or CT26 lysates prepared by two different protocols (mechanical solubilization and chemical disruption) in conjunction with CpG 1826 and Poly I:C as adjuvants. C57BL/6 mice received tumor lysate prepared by chemical disruption. Vaccinated mice were challenged with CT26 or B16F10 injected subcutaneously at the same side of the hAEC vaccination. Tumor volumes were regularly monitored and calculated by measuring tumor dimensions (L × W) with digital calipers. In the case of survival rate, mice were considered dead when the tumor surface area exceeded 225 mm^2^

In our previous experiments [10] and experiment mentioned above, we inoculated tumor cells around the vaccination site (orthotopic inoculation). In a new setting and to test cross protective vaccine effect of hAECs in cancer development, we simply used the counter side of AEC immunization for tumor challenge (heterotopic inoculation). Our results showed that mice receiving this immunization regimen developed tumors with the same size and frequency as control mice. The survival rate of these mice was not statistically different from that of control mice (Fig. 4a).

### Vaccination with hAEC lysate did not confer protection against colon cancer or melanoma

To further examine whether protection against colon cancer in hAEC-vaccinated mice drive from cross-protective immune responses, we used hAEC lysate to immunize mice before tumor inoculation. hAEC lysate was prepared either using RIPA buffer or freeze-thawing of the cells. Cell lysates were injected into the mice along with CpG and Poly I:C using a timeline mentioned above. As shown in Fig. 4b, hAEC lysate neither protected mice from colon cancer development nor extended their survival. Similarly, hAEC lysate prepared using the RIPA method did not protect mice receiving B16F10 from melanoma development (Fig. 4b). In both cancer models, however, immunization of mice with cancer cell lysate, as a preventive vaccine, considerably protected mice from cancer development and prolonged survival.

### AD-sEV isolation and characterization

**Fig. 5:**
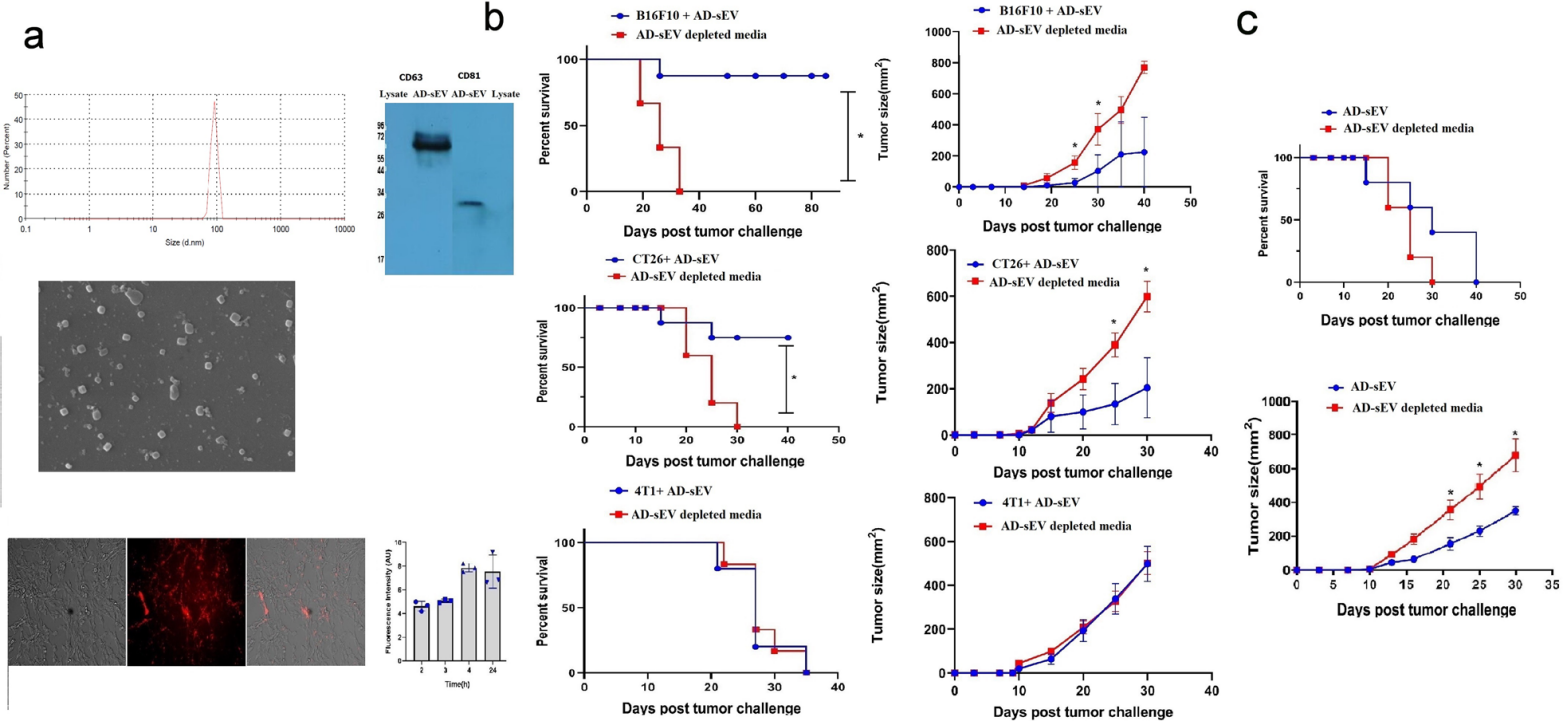
AD-sEV conferred protection and prolonged survival in mice models of colon cancer and melanoma but not in breast cancer. **a** Extracellular vesicles were isolated from hAEC and their size distribution and expression of CD81and CD63 were assessed by dynamic light scattering assay, scanning electron microscope (SEM) and Western blotting Extracellular vesicles uptake assay was performed for 2, 3, 4, and 24 h after extracellular vesicles incubation (10 μg/mL) with CT26 cells. **b** To show anti-tumor effect of AD-sEV, CT26 cells, 4T1 and B16F10 were incubated with AD-sEV (10 μg/mL) for 2 h at 37 °C and then injected subcutaneously into BALB/c and C57BL/6, respectively. Control mice received tumor cells that were incubated with extracellular vesicle-depleted cell culture medium for the 2 h. **c** CT26 tumors were established in BALB/c mice and AD-sEV were injected intratumoral when tumors became palpable. Tumor volumes were regularly monitored and calculated by measuring tumor dimensions (L × W) with digital calipers. In the case of survival rate, mice were considered dead when the tumor surface area exceeded 225 mm^2^. Error bars denote mean ± SEM. Asterisks (*) indicate statistical significant differences (*p < 0.05). AD-sEV: AEC-derived extracellular vesicles

Based on the results of aforesaid experiments, we came to the conclusion that it is not cross-protective immunity that confers protection in hAEC-immunized mice against cancer. Therefore, we isolated extracellular vesicles from hAECs and tested their potency to exert anti-cancer effects. The size of AD-sEV was about 90 ± 10 nm (Fig. 5a). To confirm the quality of AD-sEV, the expression of exosomal CD81 and CD63 was confirmed by western blotting. Specific bands of about 26 and 72 kDa were noticed for CD81 and CD63, respectively, while no band was detected in hAEC lysate (Fig. 5a). To confirm the results obtained by DLS analysis, scanning electron microscopy images of isolated EV were captured showing a relatively uniform distribution of AD-sEV size. The extracellular vesicle uptake assay showed that the extracellular vesicles are taken by CT26 cells in a time dependent manner with optimum uptake in about 4 h (Fig. 5a).

### AD-sEV conferred protection and prolonged survival in mice models of colon cancer and melanoma but not in breast cancer

To investigate whether the anti-tumor effect of hAEC vaccination is mediated by AD-sEV, CT26, B16F10, and 4T1 cancer cells were initially treated with AD-sEV or extracellular vesicles-depleted culture media for 2 h and then inoculated into the dorsal flank of mice. As shown in Fig. 5b, 60% of mice injected with AD-sEV-treated CT26 cells did not develop tumors at all. The same trend was also seen in mice injected with AD-sEV-treated B16F10; 75% of mice receiving AD-sEV-treated B16F10 showed no sign of tumor development even after 80 days post injection. As with the results obtained with whole hAEC, all mice receiving AD-sEV-treated 4T1 cells developed tumor. To determine whether AD-sEV could suppress tumor progression, 1 week after inoculation of CT26 cells, mice received three intratumoral injections of AD-sEV (1 μg) with every three days interval and tumor growth was monitored. To our results, intratumoral injection of AD-sEV significantly reduced tumor size but exerted no significant effect on overall survival (Fig. 5c).

### AD-sEV exerted cytotoxic effects on cancer cells

**Fig. 6:**
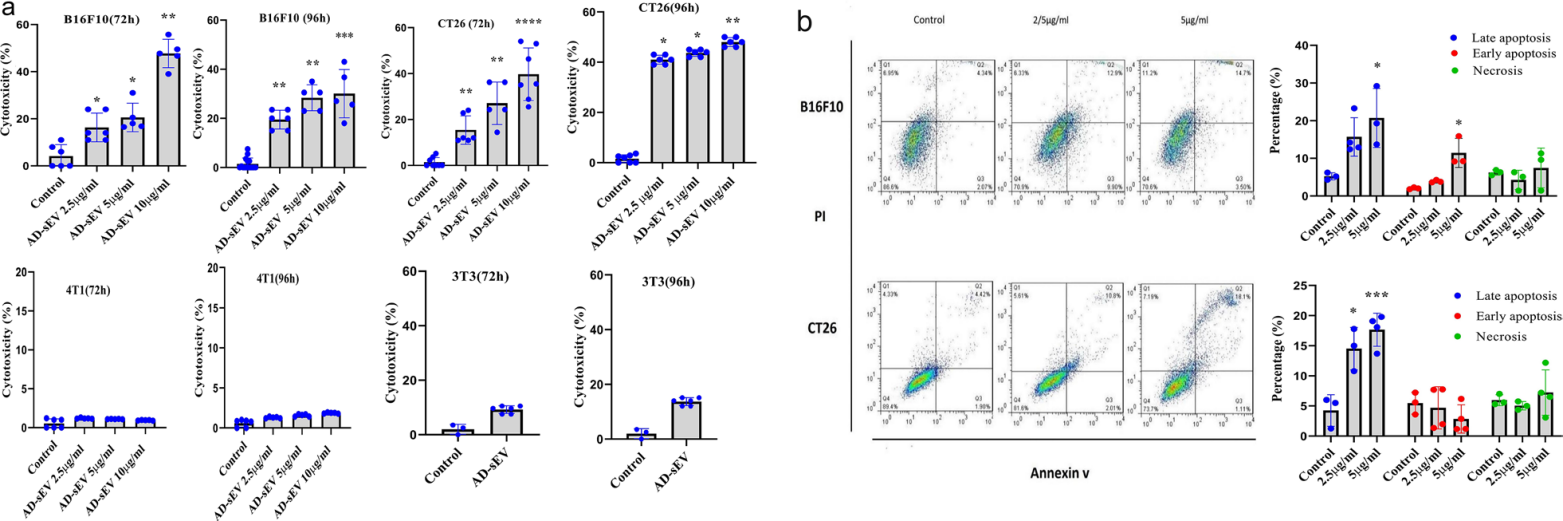
AD-sEV exerted cancer cell cytotoxicity and apoptosis. **a** B16F10, CT26, and 4T1 were incubated with different concentrations of AD-sEV for 72 and 96 h. Normal mouse fibroblasts, 3T3 cells, served as a negative cell control to evaluate the cytotoxicity of AD-sEV on non-cancerous cells. The extent of cell cytotoxicity was then assessed by MTT assay. **b** To evaluate apoptosis promoting effect of AD-sEV, B16F10 and CT26 were treated with AD-sEV (2.5 and 5 μg/mL) for 72 h. Apoptotic cell death of treated cells was detected by dual staining with Annexin V-FITC and PI followed by flow cytometric analysis. The percentage of the viable cells early apoptotic cells, late apoptosis, and necrotic cells were the determined and compared. Error bars denote mean ± SD. Asterisks (*) indicate statistically significant differences (*p < 0.05, **p < 0.01, ***p < 0.001, ****p < 0.0001). AD-sEV: AEC-derived extracellular vesicles

To evaluate the effect of AD-sEV on cancer cells in vitro, cells were incubated for 72 or 96 h with different concentrations of extracellular vesicles. AD-sEV exerted dose-dependently cytotoxic effects on CT26 cells in both time intervals examined (Fig. 6a). The extracellular vesicles did not show any cytotoxic effect on 4T1 breast cancer cells (Fig. 6a). This ineffectiveness of AD-sEV treatment on 4T1 cells was in agreement with the results of the in vivo studies; all mice in the AD-sEV-treated group developed tumor and tumor growth rate and mice survival rate was similar to that in the untreated controls. To evaluate the specificity of AD-sEV effect on cancer cells, we used normal mouse fibroblast (3T3) as a control. As shown in Fig. 6a, AD-sEV exerted a negligible effect on 3T3 cells.

### AD-sEV induced apoptosis in colon cancer and melanoma cells

An Apoptosis assay with Annexin V/PI was carried out to determine whether cytotoxicity of AD-sEV is induced by the induction of apoptosis. Incubation of CT26 and B16F10 cells with different concentrations of the purified AD-sEV for 72 h induced a sharp increase in late apoptosis in both cell lines (Fig. 6b). AD-sEV induced apoptosis in 18 ± 4 and 21 ± 8 percent in CT26 and B16F10 cells (p < 0.001). However, AD-sEV-treated cells showed the same percentage of necrotic cells as with untreated cells.

### AD-sEV boosted the cytotoxicity of splenocytes to CT26 cells

**Fig. 7:**
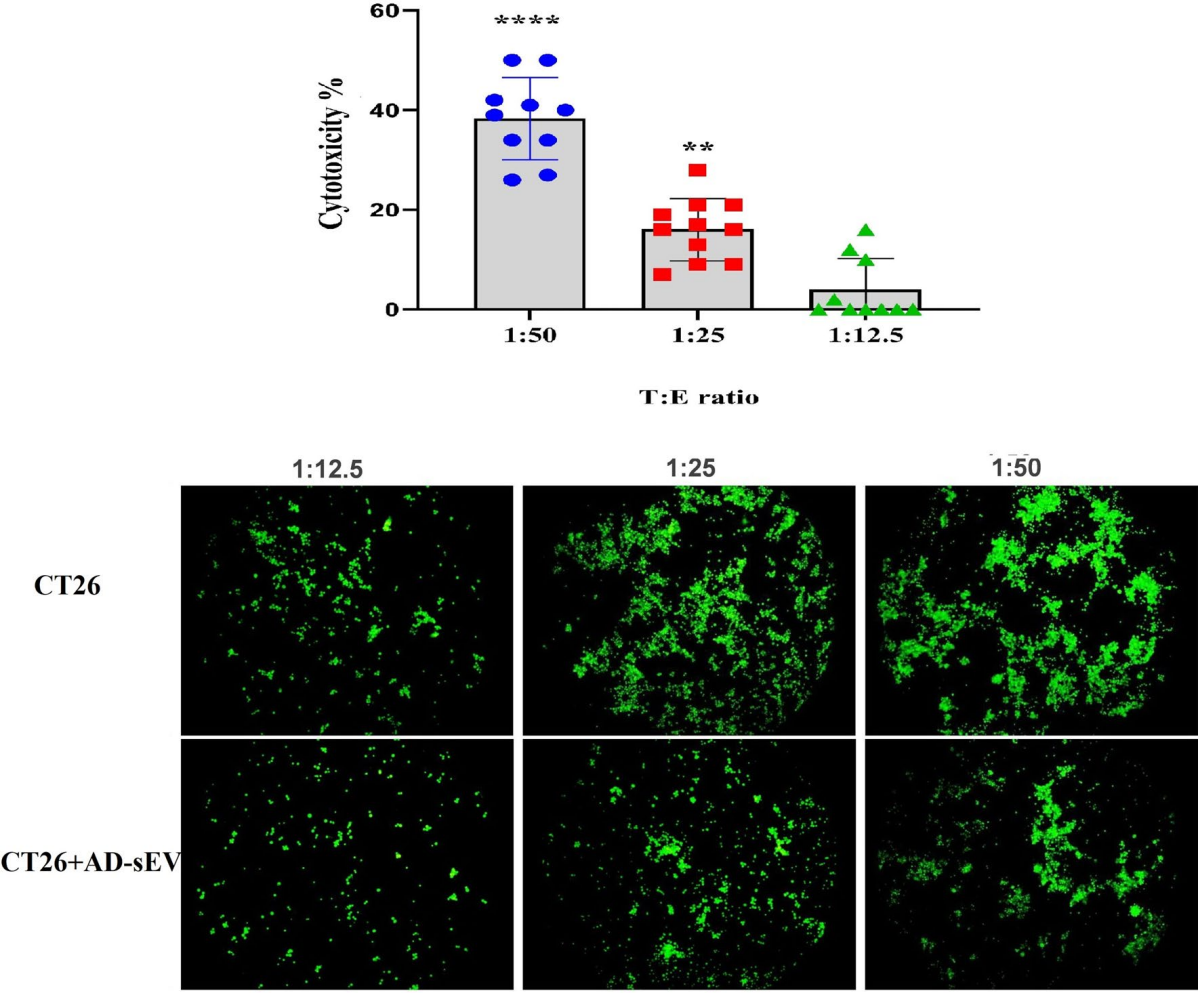
AD-sEV boosted the cytotoxicity of splenocytes to CT26 cells. BALB/c mice received either intact CT26 or CT26 cells treated with AD-sEV for 2 h before inoculation. Two weeks after cancer cell inoculation, the extent of cytotoxic responses of mice splenocytes against CT26 cells was tested and compared at different target: effector ratios by cAM assay. Each bar represents the percentage of increase in cell cytotoxicity in CT26-AD-sEV compared to CT26 group. Lower panel depicts comparative densities of cAM-labeled CT26 cells in the CT26-AD-sEV *vs.* CT26 group at different target: effector ratios. Error bars denote mean ± SD. Asterisks (*) indicate statistical significant differences (**p < 0.01, ****p < 0.0001). AD-sEV: AEC-derived extracellular vesicles

The cytotoxicity of splenocytes toward CT26 was tested in mice inoculated with either CT26 or AD-sEV-treated CT26. Cultures of different target:effector (T:E) ratios were set up and the extent of cytotoxicity was tested after 72 h using cAM assay. Analyzing the fluorescent intensity of the cultured cells showed that mice receiving AD-sEV-treated CT26 mounted significantly higher cytotoxicity toward CT26 cells at target ratios of 1:25 (p < 0.001) and 1:50 (p < 0.0001) compared to the mice inoculated with intact CT26(Fig. 7). However, this effect was diluted out in 1:12.5 T:E ratio, where no significant difference in cytotoxicity was observed between the experimental and control groups (Fig. 7).

### AD-sEV fueled Warburg’s effect

**Fig. 8:**
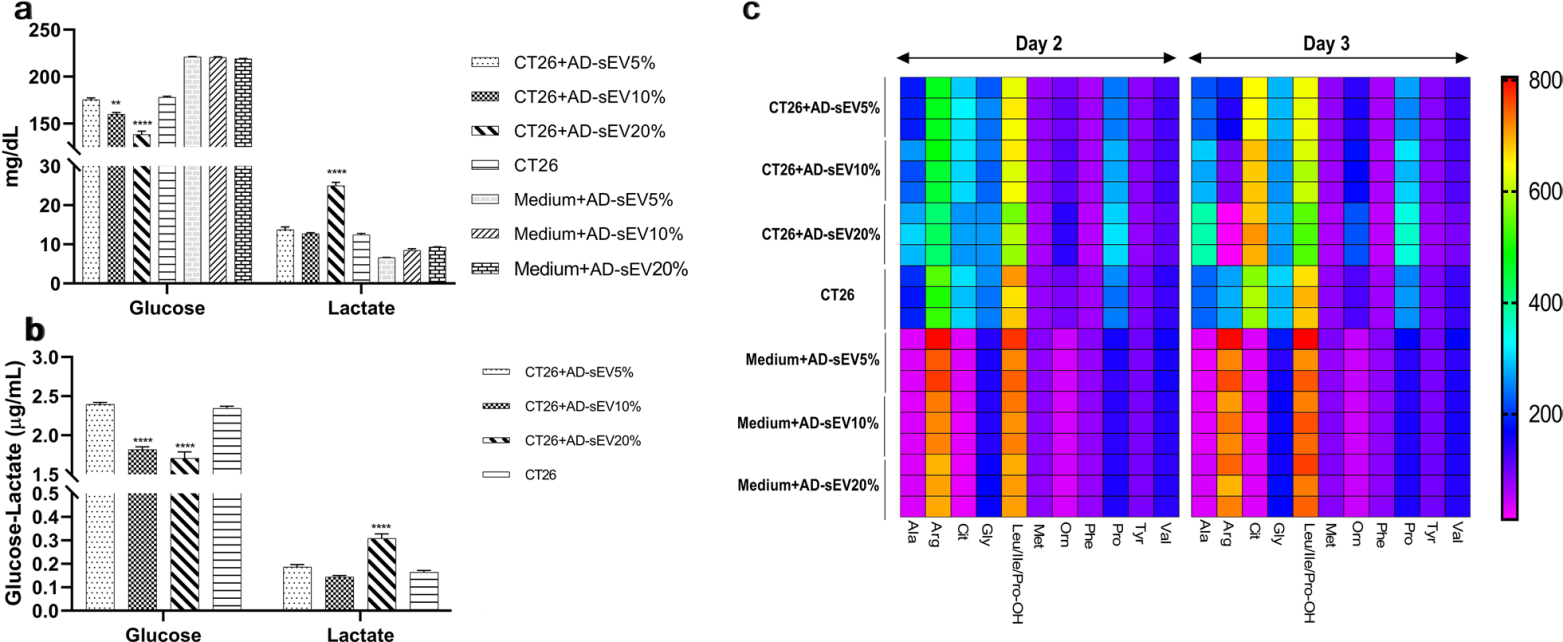
AD-sEV fueled Warburg’s effect and caused a marked reduction in Arginine concentration. **a** To test the potential effect of AD-sEV in glucose consumption and glycolysis pathway, CT26 cells were treated with different concentrations of AD-sEV and the concentrations of glucose and lactate were measured in cell culture supernatants after 72 h treatment. Untreated cells and medium alone treated with the same concentrations of AD-sEV served as controls. AD-sEV treatment caused a significant decrease in glucose concentration, while increased lactate production. **b** The levels of glucose and lactate in the above experiment were normalized to the number of living cells. **c** Effect of AD-sEV on amino acid metabolomics was measured in AD-sEV-CT26 co-culture by mass spectrometry after 48 and 72 h. The color code shows the concertation of each amino acid. Error bars denote mean ± SD. Asterisks (*) indicate statistically significant differences (**p < 0.01, ****p < 0.0001). AD-sEV: AEC-derived extracellular vesicles

To test the potential effect of AD-sEV on glucose consumption and the glycolysis pathway, CT26 cells were treated with different concentrations of AD-sEV. The results showed that after 72 h, treatment of CT26 cells with 10 and 20 µg/mL of AD-sEV caused significantly higher consumption of glucose compared to untreated cells (p < 0.01 and p < 0.0001). Consequently, 20 µg/mL of AD-sEV caused a sharp increase in lactate concentration (p < 0.0001) (Fig. 8a), Based on the fact treatment with AD-sEV caused cell death in treated cells, the levels of glucose and lactate were normalized based on the number of living cells. The results showed the same trend (Fig. 8b).

### Amino acid metabolomics showed a sharp decrease in Arginine concentration in AD-sEV-treated CT26 cells

To explore the potential effect of AD-sEV on cancer cell metabolomics, the level of amino acids in cell culture supernatants was determined by mass spectrometry. As depicted in Fig. 8c, cancer cells caused the production of Ala, Cit, Gly, Orn and Pro, while they consumed Arg, and Leu/iLeu. The concentration of other amino acids, including Met, Phe, Thy and Val remained almost unchanged in cell culture supernatants of CT26 cells compared to the medium alone. Compared with CT26 alone, cell culture supernatants of CT26 cells co-cultured with 20 µg/mL AD-sEV for 72 h, contained higher concentrations of Ala, Cit and Pro. An interesting finding was the sharp drop of Arg concentration.

### Frequency of CD4^+^/CD8^+^ T cells was not altered in the spleen of mice inoculated with AD-sEV-CT26

**Fig. 9:**
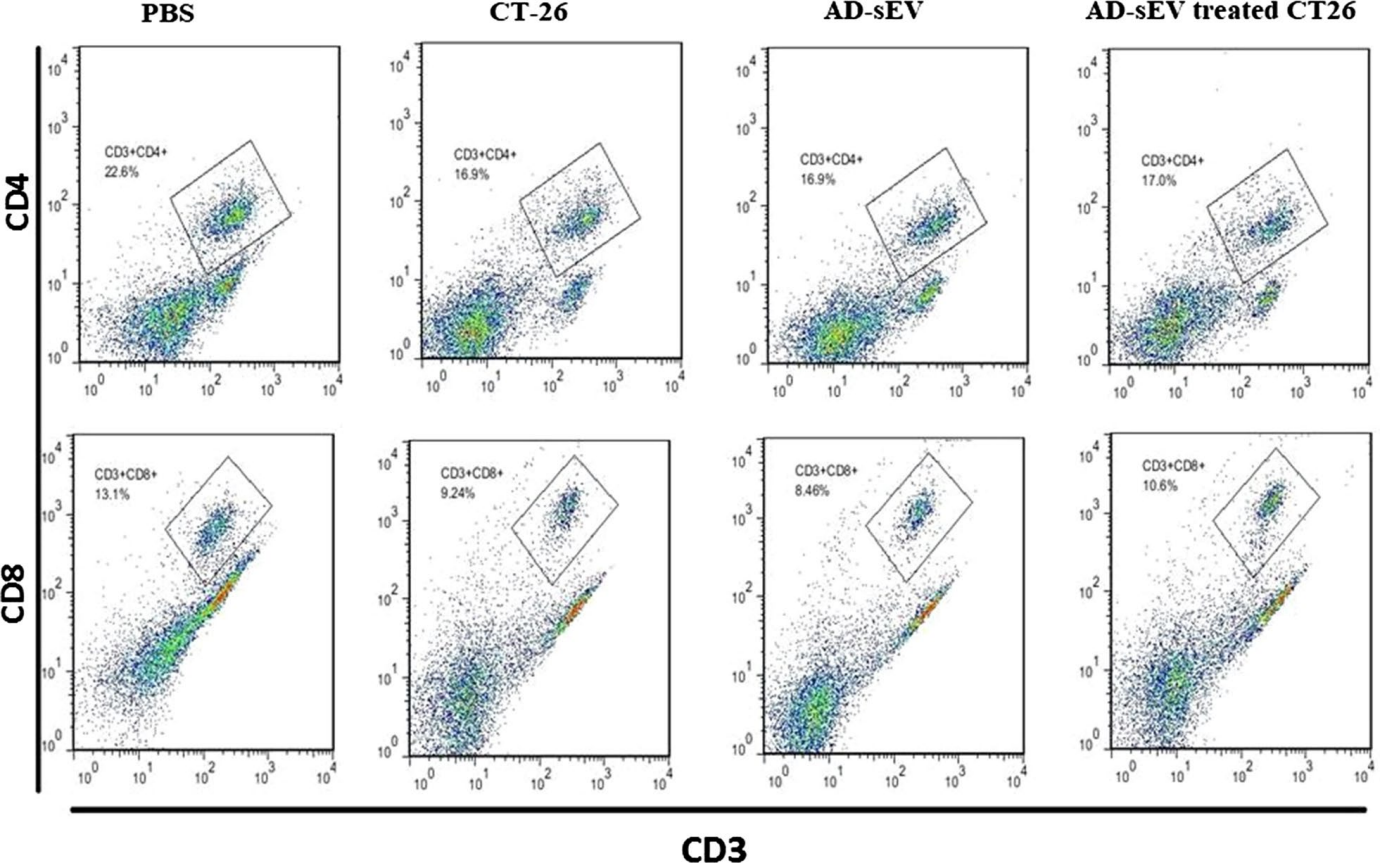
Frequency of CD4 + /CD8 + T cells was not altered in the spleen of mice inoculated with AD-sEV-CT26. The frequency of CD4 + and CD8 + T cells in splenocytes of the mice immunized with PBS, CT26, AD-sEV or AD-sEV-treated CT26 were determined by flow cytometry. Results are representative of three independent experiments

Frequency of CD4^+^ and CD8^+^ T cells was analyzed in the spleen of mice 2 weeks after inoculation with PBS, AD-sEV, CT26, or AD-sEV-treated CT26-using flow cytometry (Fig. 9). The results showed no statistical difference in the frequency of the T cells in the experimental and control groups.

## Discussion

The immunologic similarities between cancer and pregnancy and the application of fetal tissues as a preventive cancer vaccine has been proposed more than a century ago [2]. This concept has been the basis for many research experiments on the potential usefulness of immunization of stem cells from embryonic origin to hinder cancer growth or development [7, 9, 10, 43–56]. Several hypotheses have been put forth to unravel the anti-tumor effect of stem cells from embryonic origin, among them antigenic similarity and the induction of cross-protective immune responses is more fascinating. Alongside with the other researchers, we recently reported that vaccination with human AECs could effectively protect cancer development in a murine model of colon cancer [10]. We showed that hAEC could induce cancer cell cross-reactive humoral and cellular immune responses, a finding, which strengthened the concept of immunologic similarities between cancer and pregnancy. This conclusion was further substantiated by an experiment showing that immunization of mice with hAEC, which did not induce cross-reactive antibody response against 4T1, did not protect vaccinated mice to 4T1-induced breast cancer.

Subsequently, however, we obtained multiple lines of evidence showing that it is not solely antigenic similarity between cancer and AECs that confer protection after immunization of mice with these cells. First, we observed that non-fresh (cryopreserved) hAEC dramatically lost their efficacy for cross-protection against colon cancer development (unpublished data). Second, the absence of cross-reactive humoral response to B16F10 did not preclude protection against melanoma in AEC-vaccinated mice. Third, if antigen similarity between hAEC and cancer cells and induction of cross-protective immune responses are fundamental for anti-cancer effects of hAECs, one would expect that whole antigen preparation of hAEC might exert almost the same anti-cancer effect as with intact hAEC. We observed that immunization of mice with hAEC lysate, either obtained through mechanical or chemical procedures, did not exert a significant protective effect against colon cancer development, whereas immunization with live hAEC substantially inhibited cancer development. Forth and more importantly, the cancer preventive effect of hAEC immunization was totally dependent on the site of immunization and cancer cell inoculation; protection was conferred only when hAEC immunization and cancer cell inoculation were performed in the same place (orthotopic). This finding was in sharp contrast to the concept of the vaccine effect, where vaccine-induced immune responses are not affected by the location of vaccine administration. These observations clearly challenge the major contribution of cross-reactive immune responses to cancer protection in hAEC-vaccinated mice. These findings highlight the potential paracrine effect of hAEC on cancer protection.

In line with this assumption, there are some reports showing that hAEC conditioned medium inhibited the growth of breast and epithelial ovarian cancer cells via TGF β1-mediated cell cycle arrest [26, 27] or induction of apoptosis [30]. Rat AEC conditioned media showed the anti-proliferative activity on different cancer cell lines through G0/G1 cell cycle arrest. Interestingly and in line with our findings, this study showed that some cancer cells have more responsiveness to hAEC conditioned medium than the others. Moreover, while co-injection of rat AEC with B16F10 decreased significantly the tumor burden, there was no evidence of grafted AECs in the excised tumors. These results represent further proof of paracrine anti-effects of AEC [31]. In a nude mice model of human breast cancer, however, hAEC was present in the tumor site suggesting that immune cells could eliminate xenogeneic AEC [26].

The pro-apoptotic and anti-proliferative effect of CM from amniotic mesenchymal cells has also been reported, which was attributed to the down regulation in the expression of cyclins and CDKs [57]. Collectively, these results suggest that the secretome of hAEC is the main player in anti-cancer capacity of this cell type [31]. hAECs possess potent immunoregulatory properties. Previous studies have linked the production of IL6, IL10, IL1β, and TGFβ, along with prostaglandin E2 (PGE2), indoleamine 2,3-dioxygenase (IDO), and HLA-G to the anti-inflammatory properties of AEC [24]. These studies, however, do not rule out the possibility of the contribution of AEC-derived extracellular vesicles in anti-inflammatory and anti-proliferative effects of hAEC. Extracellular vesicles carry a vast array of such signaling molecules as mRNAs, miRNAs, nucleic acids, lipids, and proteins involved in intercellular communication [38, 40, 58–60]. In this regard, we hypothesized that anti-cancer effects of hAEC are mediated by AD-sEV. AD-sEV exerts various effects in the context of different conditions. In premature ovarian failure (POF) mice model, AD-sEV showed an anti-apoptotic effect in granulosa cells and protected the ovarian vasculature from damage, which was mainly mediated by miR-1246 [38]. AD-sEV were also reported to accelerate wound healing by promoting the proliferation and migration of fibroblasts through secretion of micRNAs encapsulated in EVs [40]. To the best of our knowledge, however, there is no report on the potential effect of AD-sEV on vital parameters of cancer cells. The impact of mesenchymal stem cell-derived extracellular vesicles on cancer cell proliferation and metastasis has yielded contradictory results. Several studies highlighted the supportive effect through hedgehog signalling [61], miRNA 21 and 34a [62], and the Wnt signaling pathway [63].However, some studies have indicated the anti-tumor effects through suppression of angiogenesis [64], induction of cancer cell death by TRAIL (TNF-related apoptosis-inducing ligand) delivery [65] and down-regulating phosphorylation of Akt protein kinase and up-regulating cleaved caspase-3 [66].

We found that pre-incubation of tumor cells (CT26, B16F10) with AD-sEV effectively prevented tumor development and increased survival in tumor-bearing mice. In line with this notion, we observed that AD-sEV dose-dependently induced cell apoptosis and inhibited cell proliferation of CT26, B16F10 cells. Akin to what we observed for intact hAEC, AD-sEV could also effectively prevent tumor development, reduce tumor size and prolong the survival of tumor bearing mice. These findings are in contrast with what reported earlier on anti-apoptotic and cell proliferative activities of AD-sEV and suggest that hAEC could exert different and contrasting effects depending on the context they are used. EVs carry a large set of different cargos with diverse biological functions and it is conceivable to imagine that depending on the receptors expressed on and signaling pathways active in the target cells, different biological activities occur once the cells are in contact with EVs.

The pattern of effectivity of AD-sEV on tumor development was also closely mimicked with what we observed for intact hAEC. When they are injected intratumorally, AD-sEV marginally reduced CT26- and B16F10-indeced tumor size, as we previously reported for hAEC [10].Additionally, AD-sEV did not prevent breast tumor development when incubated with 4T1 cells before tumor inoculation. This result is also consistent with the absence of protective effect of intact hAEC in the development of 4T1 breast cancer. These results clearly show that most of anti-cancer effects of hAECs are exerted through AD-sEV. Indeed, AD-sEV exerted no significant cytotoxicity against 3T3 cells, indicating that AD-sEV did not exert off target effects in normal cells. Although cross-reactive antibody responses were induced in hAEC-immunized mice against CT26, the same effectiveness of AD-sEV as with intact hAEC make the protective role of cross-reactive humoral responses insignificant. Nonetheless, it is logical to assume that apoptotic bodies released from cancer cells following treatment with AD-sEV could boost cellular immune responses through enhancement of antigen uptake by DCs and triggering antigen-specific CTL responses [67, 68]. This assumption was evident in our experiments showing that splenocytes of mice receiving cancer cells pre-treated with AD-sEV are functionally active and lyse tumor cells more efficient than those of mice receiving cancer cells alone.

Our results also clearly showed that the anti-cancer effect of AD-sEV is a function of cancer cell type. This bias of anti-cancer activity has also been reported earlier for AEC-CM [31] and explains the controversies around the extracellular vesicle effect on various tumor cells. The mechanism behind the differential effect of AD-sEV on different cancers remains to be elucidated.

To explore potential mechanisms through which AD-sEVs exert their anti-cancer effects on cancer cells, the potency of the glycolysis and amino acid metabolomics were measured in culture supernatants of AD-sEV-treated CT26 cells. Our results clearly showed that AD-sEV potentiated glycolysis pathway and fueled Warburg’s effect. Lactagenic cancer cells are characterized by increased aerobic glycolysis and excessive lactate formation, a phenomenon described by Otto Warburg. Different hypotheses have been proposed for preferential use of the glycolysis pathway by cancer cells, including rapid ATP synthesis, disruption of tissue architecture and signal transduction through ROS [69]**.** On the other hand, we showed that AD-sEV, probably through potentiation of Warburg’s effect, dramatically modulated amino acid metabolism, especially after 72 h. Such treatment resulted in a sharp decrease in arginine concentration. Interestingly, arginine deprivation is becoming a novel and promising clinical strategy for metabolism-based cancer therapy [70, 71].Increased expression of the arginine transporter CAT-1 (SLC7A1) has been reported in high-L-arginine-dependent tumors, such as breast cancer [72], colorectal cancer [73], and hepatocellular carcinoma [74] and CAT-1 silencing decreases the viability of cancer cells and induces apoptosis. Collectively, these results provide a new mechanism through which AD-sEV exerts their anti-cancer effects; fueling Warburg’s effect and running out the main amino acids necessary for cell cycle progression, arginine.

Thus, many studies have shown that hAEC cells have immune-modulating properties that suppress immune responses in animal models of autoimmune diseases [34, 39, 41, 42]. hAEC was also used in regenerative medicine to re-generate damaged tissues [18, 41, 75]. Anti-cancer effects of hAEC are also in the center of attention of many researchers. Our results propose the potential of using AD-sEV in clinical settings instead of using live hAEC, whose safety is still a matter of debate. In this regard, one approach could be targeted delivery of AD-sEV to the site of action via specific antibodies. In case of cancer, antibody drug conjugates (ADC) have been introduced as a potent tool for immunotherapy [76]. According to our results showing that AD-sEV is readily taken by tumor cells, it seems that antibody-AD-sEV-conjugate could be viewed as one modality for delivering bioactive AD-sEV to the tumor site, especially in early-stage tumors.

In conclusion, the results of the current study clearly demonstrated that although hAECs trigger cross-reactive humoral immune responses against tumor cells, these immune responses are not necessarily the major player in cancer preventive effects of hAECs; it is the AD-sEV that mediates most activity of hAEC in the prevention of cancer development through potentiation of Warburg's effect and running out arginine, as one of the main amino acids necessary for cancer cell division. Further investigations are needed to clarify the mechanism of action of AD-sEV in cancer prevention before it can be used in clinical settings.

## Data Availability

Dataset used in this study are available upon formal request.
